# In Vivo Electroporation of DNA into the Wing Epidermis of the Butterfly, *Bicyclus anynana*

**DOI:** 10.1673/031.007.5301

**Published:** 2007-10-25

**Authors:** Kyle Golden, Veena Sagi, Nathan Markwarth, Bin Chen, Antónia Monteiro

**Affiliations:** ^1^ Department of Biological Sciences, University at Buffalo, 109 Cooke Hall, Buffalo, NY 14260; ^2^ Department of Ecology and Evolutionary Biology, Yale University, P.O. Box 208106, New Haven, CT 06520-8106

**Keywords:** electroporation, *EGFP*, *Bicyclus anynana*, somatic transformation, functional genetics, Lepidoptera, wing patterns

## Abstract

The direct transfer of genes into differentiated insect tissues is a useful method of determining gene function because it circumvents the need to perform germ line transformations and of having information on tissue-specific gene promoters. Here *in vivo* gene delivery is achieved through electroporation of a reporter gene into the pupal forewing of the butterfly *Bicyclus anynana* (Butler) (Lepidoptera: Nymphalidae). Plasmids containing the coding sequence for enhanced green fluorescent protein (EGFP), driven by the *Drosophila* heat-shock promoter hsp70, were successfully expressed in epidermal cells after plasmid injection followed by electroporation and heat shock. *EGFP* expression was restricted to the vicinity of the injection and electroporation site, but the number of transformed cells varied from a few to over 5000 cells. Electroporation parameters were optimized in order to maximize the number of transformed cells while minimizing the extent of damage to the adult wing. While certain electrical parameters were well tolerated by the wing tissue, the physical damage caused by the insertion of the tungsten electrodes led to frequent disruptions of the adult wing pattern around the puncture sites. While this technique can be useful to test the correct expression of marker genes (such as EGFP) in newly build plasmids immediately following their injection, its potential use in testing the function of candidate genes in wing pattern formation is limited.

## Introduction

The use of electroporation as a genetic transformation method of broad application across species has proliferated in recent years (Moto et al. 1999; Zhang et al. 1999; Saito and Nakatsuji 2001; Haas et al. 2002; Tawk et al. 2002; Thomas 2003; Sano et al. 2003; Kunieda and Kubo 2004). Electroporation introduces foreign DNA into cells by temporarily opening pores for the DNA to enter the cell, enabling the study of regulation and function of these genes and proteins (Chang and Reese 1990). This method is of great interest because of its ability to insert DNA *in vivo* into specific tissues, including differentiated adult post-mitotic cells (Jaroszeski et al. 1999).

An *in vivo* method for candidate gene delivery into the pupal wing of the butterfly *Bicyclus anynana* is described. This approach can be used to enable the ectopic expression or disruption, via RNAi, of candidate developmental genes previously implicated in color pattern formation in butterfly wings (Brakefield et al. 1996; Keys et al. 1999; Brunetti et al. 2001; Reed and Serfas 2004; Monteiro et al. 2006), as well as the functional testing of new plasmid constructs containing candidate genes that may subsequently be used in transgenic experiments (Marcus et al. 2004).

While other somatic transformations experiments on butterfly wing tissue have successfully been performed with Sindbis and Vaccinia viruses (Lewis et al. 1999; Lewis and Brunetti 2006) they present some of limitations. Chief among them are the requirement for Biosafety Level-2 levels of laboratory safety around these viral agents, and the relatively slow growth rate of the virus in the tissues, where infected cells were usually only visible in significant clusters by 12 hours after the injection. On the positive side, when the viruses were injected into the hemocoel of the larvae, or directly into the pupal wing, there was minimal to no damage to the adult wing patterns (Lewis et al. 1999).

In the experiments described here, plasmids are electroporated directly into the wing tissue. To optimize the cell transfection efficiency during the early pupal stage, while minimizing the amount of damage that is displayed on the adult wing, the DNA sequence for enhanced green fluorescent protein (EGFP) was used as a reporter. The method was also tested using a new plasmid construct containing a candidate developmental gene, *Decapentaplegic* (*Dpp*), fused to *EGFP*.

## Materials and Methods

### Plasmid preparation

The piggyBac constructs *pBac {3xP3-DsRed, hsp70-EGFP}* (Ramos et al. 2006) and *pBac {3xP3-EGFP, hsp70-Dpp-EGFP}* (B. Chen et al., in prep.) containing the DNA sequence for the reporter protein enhanced green fluorescent protein (EGFP), were introduced through electroporation into somatic cells of the butterfly *B. anynana* and their ability to be expressed was analyzed. Both plasmids used the heat-shock *Hsp70* promoter from *Drosophila* to drive gene expression and the latter plasmid contained the *Decapentaplegic* (*Dpp*) gene from *Drosophila* fused to EGFP (Teleman and Cohen 2000). The plasmids were amplified in JM109 *Escherichia coli* cells and isolated using Qiagen Hispeed Plasmid Maxi kit. The DNA concentration used ranged from 550 to 940ng/µl. For a trial of 10 pupae, 20 µl of plasmid and 0.5µl of McCormick blue dye was mixed together. The dye was used to visualize the injection. For control injections, 20 µl of water were added to 0.5 µl of dye. For trials using the transfection reagent Flyfectin™ (Oz Biosciences, catalog number FF51000), a 2:3 ratio of DNA to Flyfectin™ was used, reducing the concentration of DNA to a range of 550 – 570 ng/µl. In trials where water was substituted for Flyfectin™, a 2:3 ratio of DNA to water was used, thus keeping the concentration of the DNA constant.

### Plasmid injection into pupae

**Figure 1.**
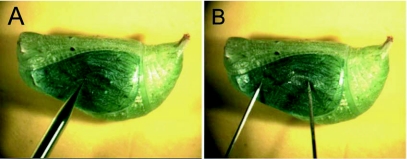
Injection and electroporation method. A) Injection of blue-colored DNA plasmid into the wing epidermis of B. anynana using a pulled glass micropipette. B) Electroporation of the wing epidermis using fine tungsten needles connected to an electrical power supply.

Pupae between the ages of 3 to 19.5 hours old were injected using a pulled glass microcapillary (TW120F-3, World Precision Instruments, www.wpiinc.com), attached to a Picospritzer III microinjection apparatus (www.parker.com), containing approximately 5 µl of the plasmid mixes mentioned above. A suitable location, generally in the middle of the forewing of the pupa, was selected for injection (Figure 1a). The microcapillary was inserted through the cuticle of the pupa at a tangential angle. Delivery of the plasmid occurred through a variable number of pulses of pressure where approximately 2 µl of plasmid was injected per pupa. While discharging bursts of pressure, the microcapillary was slowly moved up and down in order to distribute plasmid at variable depths within the first 1–2 mm of the surface of the pupa. Control injections with blue colored water, instead of DNA, were performed throughout the experiment.

### Plasmid electroporation

Once all of the pupae were injected with the plasmid, the DNA was electroporated into cells using a BTX Electro Square Porator ECM 830 (www.btxonline.com). Two fine tungsten needles (50 mm long, 0.25 mm diameter, 500134, World Precision Instruments) were used to penetrate the cuticle of the pupa. The needles were positioned approximately 2.5 mm apart (Figure 1b shows correct distance between needles, but not the correct angle of needle insertion), and inserted into the cuticle at a depth of approximately 1 mm. Electroporation conditions varied in pulse number (3–10), voltage (20V-160V), pulse length (100 µs - 1.5 seconds) and interval (100 – 500 ms) between pulses. Immediately following electroporation the pupae were heat shocked for 2 - 5.5 hours at 39°C to induce *EGFP* expression via the *Drosophila* heat-shock promoter . Control pupae, injected with water and dye, were electroporated and heat-shocked across the same range of parameters as the pupae injected with DNA.

### Wing preparation and visualization of EGFP

Wing extractions were performed 1.5 – 27 hours following the heat shock in phosphate buffered saline solution at 4°C. An incision was made around the entire cuticle of the forewing using a scalpel to detach the wing with the adjoining cuticle from the rest of the pupa. The wing was then separated from the cuticle with fine curved forceps and incubated for 30 minutes on ice in wells containing 0.5 µl of blue-fluorescent reactive dye (LIVE/DEAD Reduced Biohazard Cell Viability Kit#4, Molecular Probes, www.invitrogen.com) and 500 µ buffered saline. This dye can only penetrate dead or membrane compromised cells and was used to estimate the amount of cell death around the electroporation sites. After the incubation period, the wings were washed twice with 500 µ of buffered saline. The wings were further incubated for 15 minutes in 3.7% formaldehyde solution (from a 37% stock solution diluted in buffered saline) at room temperature. Next, the wings were washed with 500 µ of buffered saline and mounted on a slide in 15 µ Slow Fade® Light Antifade Kit (Molecular Probes). EGFP and the blue fluorescent reactive dye were observed under a *Leica* DMIRE II fluorescent microscope at 100x and 2OOX using appropriate DAPI and FITC filter sets.

### Emerged butterflies

Pupae that were allowed to emerge following the heat shock, were placed in small mesh cages inside an insectary kept at 27°C and 80% relative humidity until adult butterflies emerged 7 days later. Adult butterflies were frozen at -2O°C and forewings were subsequently examined for any damage or color pattern alteration.

## Results and Discussion

### Electroporation efficiency

**Figure 2.**
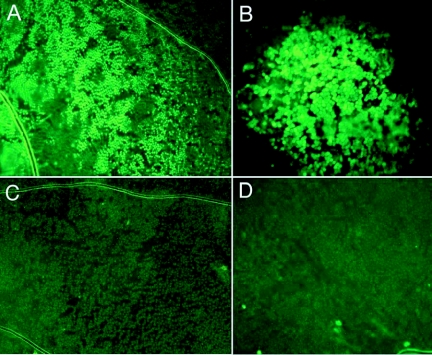
Wing epidermal cells expressing *EGFP* after plasmid injection followed by electroporation. A) 3 pulses of 80 V, o.1 ms pulse duration, and 100 ms pulse interval (100X magnification). B) 3 pulses of 80 V, 1 second pulse duration, and 500 ms pulse interval (200X magnification). C) and D) Epidermal cells of wings treated in the same way as in A) and B) but injected with blue colored water.

Using a variety of different electroporation parameters a plasmid containing the coding sequence of *EGFP* was introduced into the epidermal cells of the pupal forewing of *B. anynana* and induced the cells to produce the fluorescent protein (Figure 2; Table 1). None of the control pupae injected with water displayed any green fluorescence in the epidermis. The use of Flyfectin reduced the clarity of visualization of EGFP expression, possibly due to the lipid composition of this product. The highest transfection rate was obtained when DNA was mixed with Flyfectin, and the electroporation was done with 3 pulses at 80 V, with 1 second pulse length, and 500 ms interval between pulses, followed by a heat shock of 3.0 hours (Table 1). Of the pupae injected under these conditions 68% expressed some level of EGFP. Averaging across electroporation conditions (rows 5–10 in Table 1), Flyfectin did not have a significant effect on increasing the number of pupae expressing some level of EGFP using a Chi-square test comparing 29 out of 60 animals that expressed EGFP with Flyfectin versus 20 out of 59 without Flyfectin. The efficiency of gene expression was found to be dependent mostly on the pulse length. A pulse length of 1 second was found to result in significantly higher number of transfected individuals relative to pulses of 0.5 seconds or 1.5 seconds, respectively [Chi-square between rows 5+6 (0.5s) and 7+8 (1s), p<0.001, between rows 7+8 (1s) and 9+10 (1.5s) p<0.025; Table 1]. Across most experiments, cellular expression of EGFP varied widely from a few epidermal cells to over 5000 cells, all centered near the site of injection. Variation within any of the experimental conditions, however, was usually much larger than variation across conditions (see last column of Table 1), and there was no clear correlation between pulse length and number of transformed cells. In one of the shorter pulse length experiments (o.1 ms), for example, the largest number of transfected cells was obtained (Figure 2a). This suggests that there are other, yet unidentified, and possibly stochastic factors that play a role in cell transformation.

**Table 1.**
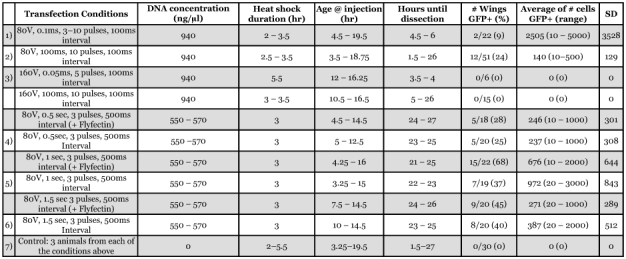
Effect of different electroporation conditions on the expression of the plasmid *pBac {3xP3-Dsred, hsp70-EGFP}* in the pupal forewing of *Bicyclus anynana*. Numbered rows (1–7) can be compared with similarly marked rows in Table 2.

Voltage, however, appears to be critical in obtaining successful plasmid transfection. Initial trials used a range of voltages (from 20 V to 80 V) with 3 pulses 100 µs in length. The only voltage condition that led to some *EGFP* expression was 80 V. We decided to concentrate our subsequent trials using this and higher (160 V) voltages and to vary pulse length and other parameters to optimize transfection rate. In all trials, using an average of 10 individuals per trial, some proportion of individuals showed expression of EGFP at 80 V. However, when voltage was increased to 160 V, we were no longer able to observe *EGFP* expressing cells (Table 1).

### EGFP-Dpp fusion construct

A new plasmid construct, *pBac [3xP3-EGFP, hsp70-Dpp-EGFP]* (B. Chen et al., in preparation) containing a candidate developmental gene from *Drosophila, Decapentaplegic*, fused to EGFP (Teleman and Cohen 2000) was tested in *Bicyclus* using the optimal electroporation conditions observed in previous experiments (3 pulses at 80 V, 1 second pulse length, and 500 ms interval between pulses). Approximately 100 cells expressing *EGFP* were observed both in the presence and absence of Flyfectin, confirming that the new construct is producing a fluorescent protein. It remains to be tested whether this protein is functional. We are planning to produce transgenic lines of butterflies with this construct to test *Dpp*'s role in wing pattern formation in *Bicyclus*.

### Survival and tissue damage

Specific electroporation conditions have a dramatic effect on survival and on the appearance of the adult butterfly. Despite minimal cell death observed immediately after the wings were extracted by means of a live/dead viability stain, butterflies that survived the treatment had a wide range of injuries, including ectopic eyespots, holes in the wing that matched the electroporation sites, ectopic black or gold scales centered around the holes in the wings, and wings that did not expand upon emergence or that were still attached to the pupal case (Table 2). At 80 V and using 3 pulses and 500 ms interval between pulses, the survival rate did not depend on pulse length (Table 2 comparing rows 4, 5, and 6; Chi-square p>0.O5). This was also the case for animals injected with water instead of DNA (rows 7, 8, 9; Chi-square p>0.05). The presence of DNA, versus water, did not alter the proportion of animals that survived the 0.5, 1, or 1.5 second pulse treatments. The combination of higher voltages (160 V) with short pulse lengths (0.05 ms), and lower voltages (80 V) with longer pulse lengths (1.5 s) has similar effects on mortality. The effect of Flyfectin on pupal survival was not tested as its effect on transformation rate was considered small to justify its use in future experiments.

**Figure 3.**
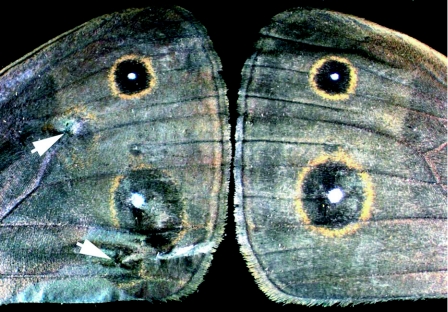
Adult wing pattern of pupae treated with 3 pulses of 80 V, 1 second pulse length, and 500 ms between pulses, injected with blue colored water, showing ectopic eyespots at positions where the electrodes were inserted (arrows). Control wing (left wing of the same animal) is also shown for comparison.

From the group of butterflies that survived the treatment, approximately 30–40% had wings that did not expand properly and most of the remaining animals had either small holes or ectopic black and gold scales around the areas where the electrodes were inserted (Table 2). These effects were visible also for control animals injected with water (Figure 3). Slight variations in the exact location where the electroporation needles were inserted may explain some of the outcomes of these experiments. Wings pierced by an electrode placed close to the wing hinge (as illustrated in Figure 1b) may have had less chance of properly expanding relative to wings that were pierced closer to the wing margin (as illustrated in Figure 3). The appearance of ectopic scales was expected because previous research has shown that inducing injury to pupal wings in the first 24 hours of pupal development can differentiate rings of black and gold colors on the adult wing (Monteiro et al. 2006; Monteiro et al. 1994).

**Table 2.**
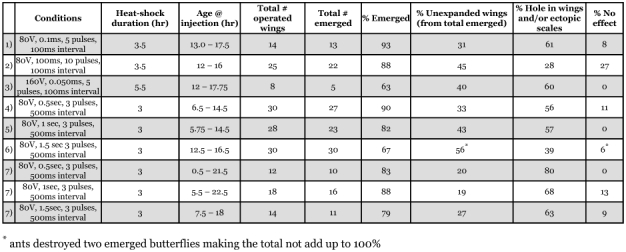
Effect of electroporation conditions on pupal mortality, and on the adult wing of Bicyclus anynana. None of these experiments used Flyfectin. Animals in rows number 1–6 were injected with DNA whereas animals in row number 7 were injected with water.

The set of conditions tested that optimize transfection rate are not the same as those that minimize wing damage to the adult. The optimal conditions observed for transfection were 3 pulses of 80 V, 1 second pulse length, and a 500 ms interval between pulses using Flyfectin, whereas the optimal conditions for minimizing damage on emerged butterflies were 3 pulses of 80 V, 100 ms pulse length, and a 100 ms interval between pulses. The optimal set of conditions that led to maximal transfection efficiency with minimal effect on the wing and the wing pattern was 10 pulses at 80 V, 100 ms pulse length, and 100 ms interval between pulses with a DNA concentration close to 1mg/µl (no Flyfectin was used in these experiments). Optimal conditions were estimated by multiplying the “% Wing GFP +” column values in Table 1 by the corresponding “% no effect” values in Table 2, in rows with comparable sets of conditions, and determining the rows with highest product value. In all there was a 6.5% probability of observing some effect of a test candidate developmental gene that is not confounded with the effect of the operation.

From these analysis we conclude that plasmid injections followed by electroporation may still need further improvement when being used to visualize the effect of transgenes on the adult wing with minimal confounding effects from the operation electroporation of plasmid DNA. This technique, however, may be used effectively to test the functionality of new plasmid constructs, especially if these constructs contain a fluorescent marker protein (at least 1 in 4 individuals will show some level of *EFGP* expression). Experiments aiming to optimize other parameters that were kept constant or varied within a narrow range of values (DNA concentration, voltage, pulse number, and interval between pulses) can also be tested in future to obtain a set of electroporation conditions that maximize transfection rate and minimize wing damage in the adult. In particular, a higher number of pulses, which do not seem to increase wing damage (compare rows 1 and 2 in Table 2), could be tested to try obtain a higher transfection efficiency. Also, pupal age, which was variable at the time of injection, could be an additional factor that influences transformation efficiency, mortality, and/or probability of wing damage. Perhaps the most important modification should be to try and insert the electrodes in areas of the pupae that are adjacent, but not on top of the pupal wing tissue.

### Comparison with previous studies on electroporation of insect tissues

Our study is not the first that attempts to electroporate plasmids into the insect wing epidermis. Thomas (2003) also attempted similar experiments using *Bombyx mon* embryos and dissected larval wings and ovaries. He found that 2 out of 16 wings expressed some number of cells expressing Lac Z using 5 pulses of 250 V, 50 ms pulse duration, and 1 s between pulses. These two wings had an average of 36 “spots” of positive cells. No determination of survivorship or tissue damage was determined for these experiments as the tissues tested were first removed from the animal.

Electroporation conditions used for gene transfer into other insect tissues, in particular brains of adult honeybees and of *Bombyx mori* (Kunieda and Kubo 2004; Moto et al. 1999), were also different from the optimal conditions determined in our experiment. Five pulses of a shorter pulse length of 50 ms combined with lower voltages (50/60 V) were used in both these experiments. In the honeybee experiment, transformation efficiency increased gradually with voltage but so did mortality. At 50/60 V there was approximately 50% mortality of the adult bees. *Bombyx* brains were electroporated *in vitro* and no mortality rates were assessed. The pulse length and number of pulses were kept constant in both these experiments.

In butterfly wings, neither 80 V nor 160 V led to high mortality (Table 2), but 160 V resulted in no visible transformation, and both of these voltages, in combination with longer pulses, caused slightly higher occurrence of wing damage. These results suggest that depending on the type of tissue or organism being electroporated, specific optimal electroporation conditions need to be developed.

Electroporation of plasmids using some of the conditions tested here will be sufficient to transfect fairly large populations of epidermal cells in developing pupal wings. This technique, however, leads to a large proportion of adults carrying distortions to the wings and the wing patterns. Future developments of this technique should continue to try and dissect apart the effect of the transgene being tested from the possible wounding effects of the operation on the adult wing. A promising new alternative to electroporation and viral somatic transfection that could also be attempted would be the use of sonoporation of plasmids into insect tissues (Lee et al. 2005).

## Abbreviations

EGFP -: enhanced green fluorescent protein
